# A First Report on [^18^F]FPRGD_2_ PET/CT Imaging in Multiple Myeloma

**DOI:** 10.1155/2017/6162845

**Published:** 2017-07-27

**Authors:** Nadia Withofs, François Cousin, Bernard De Prijck, Christophe Bonnet, Roland Hustinx, Sanjiv S. Gambhir, Yves Beguin, Jo Caers

**Affiliations:** ^1^CHU of Liege, Nuclear Medicine and Oncological Imaging Division, Medical Physics Department, Liege, Belgium; ^2^CHU of Liege, Department of Clinical Hematology, Liege, Belgium; ^3^Molecular Imaging Program at Stanford (MIPS), Radiology Department, Stanford University, Stanford, CA, USA

## Abstract

An observational study was set up to assess the feasibility of [^18^F]FPRGD_2_ PET/CT for imaging patients with multiple myeloma (MM) and to compare its detection rate with low dose CT alone and combined [^18^F]NaF/[^18^F]FDG PET/CT images. Four patients (2 newly diagnosed patients and 2 with relapsed MM) were included and underwent whole-body PET/CT after injection of [^18^F]FPRGD_2_. The obtained images were compared with results of low dose CT and already available results of a combined [^18^F]NaF/[^18^F]FDG PET/CT. In total, 81 focal lesions (FLs) were detected with PET/CT and an underlying bone destruction or fracture was seen in 72 (89%) or 8 (10%) FLs, respectively. Fewer FLs (54%) were detected by [^18^F]FPRGD_2_ PET/CT compared to low dose CT (98%) or [^18^F]NaF/[^18^F]FDG PET/CT (70%) and all FLs detected with [^18^F]FPRGD_2_ PET were associated with an underlying bone lesion. In one newly diagnosed patient, more [^18^F]FPRGD_2_ positive lesions were seen than [^18^F]NaF/[^18^F]FDG positive lesions. This study suggests that [^18^F]FPRGD_2_ PET/CT might be less useful for the detection of myeloma lesions in patients with advanced disease as all FLs with [^18^F]FPRGD_2_ uptake were already detected with CT alone.

## 1. Background

The introduction of efficient and less toxic treatments caused a paradigm shift in the management of multiple myeloma (MM) towards an earlier diagnosis and treatment [1, 2]. To detect early signs of bone disease and to identify those patients for whom treatment is needed, highly sensitive imaging techniques are required. Positron emission tomography combined with computed tomography (PET/CT) using [^18^F]fluorodeoxyglucose ([^18^F]FDG) has already proven to be a sensitive technique for the detection of metabolically active MM lesions and was recently incorporated in the diagnostic work-up of MM by the International Myeloma Working Group (IMWG) accordingly [3].

Alternatively, the ^18^F-FB-mini-PEG-E[c(RGDyK)]_2_ ([^18^F]FPRGD_2_), a validated radiopharmaceutical with high binding affinity for integrin *α*_*v*_*β*_3_, seems attractive for the detection of MM lesions [4–8]. The RGD-based radiopharmaceuticals were initially developed to accelerate the development of therapies targeting integrin *α*_*v*_*β*_3_ [9]. The high expression of integrin *α*_*v*_*β*_3_ by activated endothelial cells during angiogenesis aroused keen interest in RGD-based radiopharmaceuticals for imaging of tumor angiogenesis [10, 11]. Nevertheless, the integrin *α*_*v*_*β*_3_ is not solely expressed by activated endothelial cells; it can be overexpressed by many types of cancer cells, regulating cell survival, metastases, and drug resistance [12]. In the case of myeloma, the integrin *α*_*v*_*β*_3_ is expressed by activated endothelial cells but it can also be overexpressed by myeloma tumor cells and other cell types of the tumor microenvironment such as osteoclasts [13–17]. Our group previously studied the use of [^18^F]FPRGD_2_ in rectal and renal cancers, where a correlation between integrin *α*_*v*_*β*_3_ expression and tracer uptake was shown [7, 8]. Since multiple players within the myeloma microenvironment express the integrin  *α*_*v*_*β*_3_, we hypothesized that [^18^F]FPRGD_2_ PET/CT could be an effective imaging technique for the detection of myeloma lesions.

The combination of [^18^F]NaF and [^18^F]FDG for PET/CT is another strategy to improve the detection of bone metastases and was first introduced by Iagaru et al. [18, 19]. The rationale for the use of both [^18^F]NaF, allowing the detection of bone metastases with bone formation, and [^18^F]FDG, enabling the detection of metastases with increased rate of glucose metabolism, was to improve the sensitivity for detecting metastatic lesions. A prospective clinical trial evaluating combined [^18^F]NaF and [^18^F]FDG for PET/CT in patients with MM is currently under investigation (EudraCT 2013-004807-38), aiming at comparing its capacity to detect MM lesions with the capacities of magnetic resonance imaging, CT alone, and whole-body X-rays [20].

The current observational study was set up to assess the feasibility of [^18^F]FPRGD_2_ PET/CT to identify myeloma lesions. Secondarily, the detection rate of [^18^F]FPRGD_2_ PET/CT was compared to CT alone. Additionally, [^18^F]FPRGD_2_ PET/CT images were compared to combined [^18^F]NaF/[^18^F]FDG PET/CT images, available for those patients that were also included in the above-mentioned trial [20].

## 2. Materials and Methods

Patients with newly diagnosed or relapsed MM were prospectively included. This study was registered as EudraCT #2013-004807-38 and was approved by the Ethics Committee of the academic hospital (CHU of Liege). All subjects provided written informed consent for this study.

The radiosynthesis of [^18^F]FPRGD_2_ was performed as previously reported and in compliance with current good manufacturing practice regulations [5, 7]. The mean (±standard deviation) injected mass of the active pharmaceutical ingredient was 11.1 *μ*g (±1.6 *μ*g) [7].

Every patient underwent whole-body (WB) scans, from vertex to toes, using [^18^F]FPRGD_2_ PET/CT and combined [^18^F]NaF/[^18^F]FDG PET/CT (median delay between scans: 4 days; range: 3–5 d). PET/CT scans were acquired in a Gemini TF scanner after injection of 296 ± 9 MBq [^18^F]FPRGD_2_ (median uptake time: 62 min) or 133 ± 6 MBq [^18^F]NaF and 242 ± 27 MBq [^18^F]FDG (median delay between [^18^F]FDG and [^18^F]NaF injections: 2 min and uptake time: 66 min). All patients fasted for 6 h prior to radiopharmaceutical injection (glycemia < 120 *μ*g/ml in all patients). A low dose CT (3 mm slice thickness; 120 kV and 50 to 80 mAs depending on patient's weight) followed by the PET emission scan of 90 seconds per bed position was performed.

The PET/CT images were reviewed by 2 experienced nuclear medicine physicians and 2 radiologists to detect focal lesions (FLs) and/or diffuse bone marrow involvement. Areas of tracers' uptake corresponding to degenerative changes were excluded. Focal areas of increased uptake, regardless of the presence of bone abnormality on CT images, and hypoactive FLs with underlying bone destruction on CT images and suspected of being associated with myeloma lesions were considered PET MM FLs. The FLs were classified according to their location in 7 regions of the body: pelvis, skull, superior limbs, inferior limbs, spine, ribs, and one location including the sternum, scapula, and clavicles. A 1.2 ml volume of interest was drawn in the focal area of radiopharmaceutical's uptake to estimate the maximum standardized uptake value (SUV_max_). The maximum diameter of the osteolytic lesions, when present, was also measured. The results are presented as means ± standard deviation (SD).

## 3. Results

**Figure 1:**
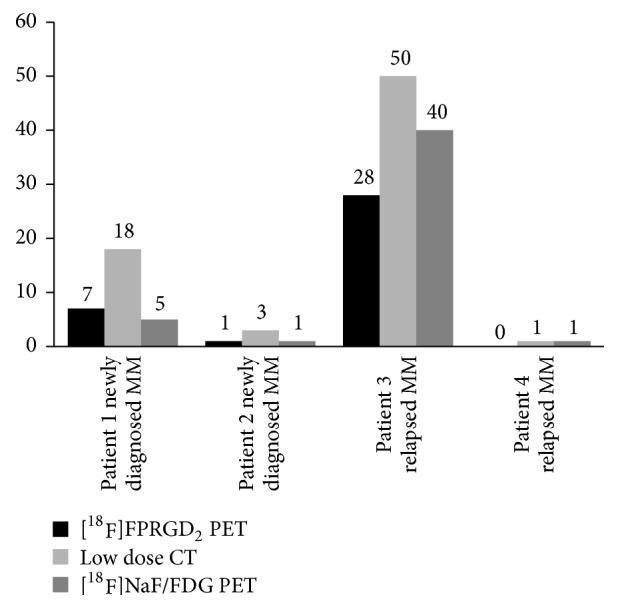
Detection rate of osteolytic FLs of CT, [^18^F]NaF/FDG PET/CT, and [^18^F]FPRGD_2_ PET/CT per patient (*n* = 4) and overall.

**Figure 2:**
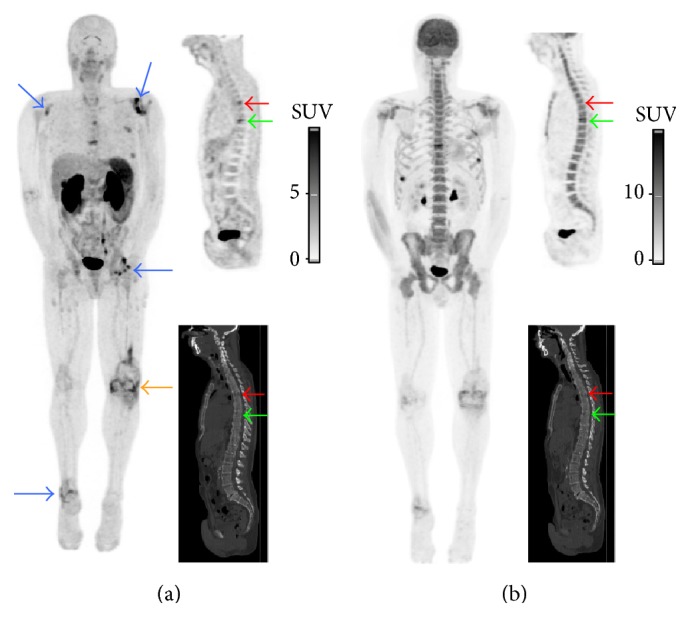
[^18^F]FPRGD_2_ and [^18^F]NaF/[^18^F]FDG PET/CT images of patient #1 with newly diagnosed MM. The [^18^F]FPRGD_2_ PET/CT images ((a) maximum intensity projection, MIP, and sagittal slices) show two spinal FLs with [^18^F]FPRGD_2_ uptake: one in the vertebral body of T5 corresponding to a mixed lesion on CT images ((a) red arrows) and a pathologic fracture of T8 ((a) green arrows). The [^18^F]NaF/[^18^F]FDG PET/CT images ((b) MIP and sagittal slices) show [^18^F]NaF/[^18^F]FDG uptake in T8 ((b) green arrows) but not in T5 ((b) red arrows). In addition, [^18^F]FPRGD_2_ uptake was also observed in glenohumeral, left hip, and right ankle joints ((a) blue arrows) as well as in the left total knee arthroplasty ((a) orange arrow). The observation of [^18^F]FPRGD_2_ uptake in musculoskeletal disorders has already been published [6].

**Figure 3:**
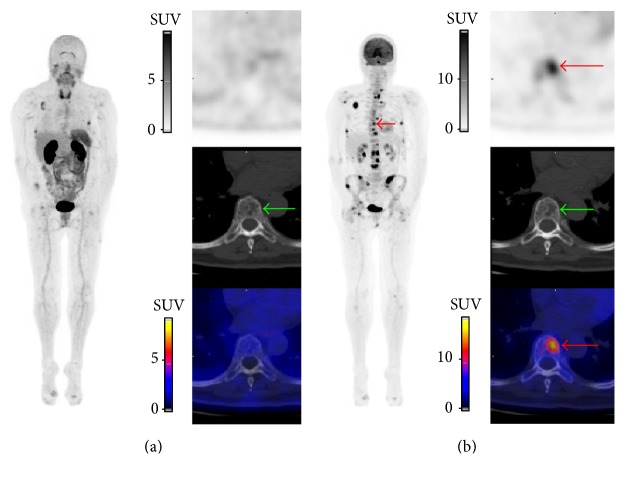
[^18^F]FPRGD_2_ PET/CT (a) and [^18^F]NaF/[^18^F]FDG PET/CT (b) images of patient #3 with MM at time of relapse, more than 4 years after diagnosis and end of treatment. The number of osteolytic FLs with [^18^F]FPRGD_2_ uptake (*n* = 28) was far lower than with [^18^F]NaF/[^18^F]FDG uptake (*n* = 40). The green arrows point at an osteolytic FL of T9 showing high [^18^F]NaF/[^18^F]FDG uptake ((b) red arrows; SUV_max_ 10.2) but no focal [^18^F]FPRGD_2_ uptake ((a) SUV_max_ 1.8).

Four patients with MM were included, *n* = 2 with newly diagnosed MM and *n* = 2 with relapsed MM (Table 1). Based on the low dose CT images, the pattern of bone marrow involvement was focal (*n* = 2) or combined diffuse and focal (*n* = 2). Per patient, ≤3 FL (*n* = 2) or >10 FLs (*n* = 2) were detected. No extramedullary disease was detected. Overall, 81 FLs were detected with PET/CT with underlying bone destruction on CT images (*n* = 72; 89%) or fractures (*n* = 8; 10%; vertebra *n* = 5; rib *n* = 3) and one FL (1%) detected with [^18^F]NaF/[^18^F]FDG PET in the femur did not show any abnormality on CT images. Overall, the detection rate of [^18^F]FPRGD_2_ PET was lower than [^18^F]NaF/[^18^F]FDG PET, whatever the FL location, and the mean uptake (SUV_max_) of [^18^F]FPRGD_2_ was overall lower than [^18^F]NaF/[^18^F]FDG (Table 2). Out of the 72 osteolytic FLs detected with the CT of the PET, only 50% (36/72) showed [^18^F]FPRGD_2_ uptake (Figure 1). Nonetheless, in one patient with newly diagnosed MM (Figure 1: patient #1), five FLs showed [^18^F]FPRGD_2_ uptake but no [^18^F]NaF/[^18^F]FDG uptake (Figure 2). In patient # 2 (Figure 1), both [^18^F]FPRGD_2_ and [^18^F]NaF/[^18^F]FDG PET/CT detected one rib osteolytic FL, while 2 additional osteolytic FLs were detected with CT. In patient #3 (Figure 1), the detection rate of [^18^F]FPRGD_2_ PET was much lower than [^18^F]NaF/[^18^F]FDG PET (Figure 3). In patient #4 (Figure 1), [^18^F]FPRGD_2_ PET/CT overlooked one 5 mm osteolytic FL of the cortical bone of a femur that was detected with [^18^F]NaF/[^18^F]FDG PET/CT. In the contingency Table 3, the obtained results in patients with newly diagnosed disease are compared to those of patients with relapsing disease. [^18^F]FPRGD_2_ positive lesions without concomitant [^18^F]NaF/[^18^F]FDG uptake were observed in one patient with newly diagnosed disease, while patient #3 (with a disease relapse) showed [^18^F]NaF/[^18^F]FDG positive lesions without [^18^F]FPRGD_2_ uptake.

## 4. Discussion

**Figure 4:**
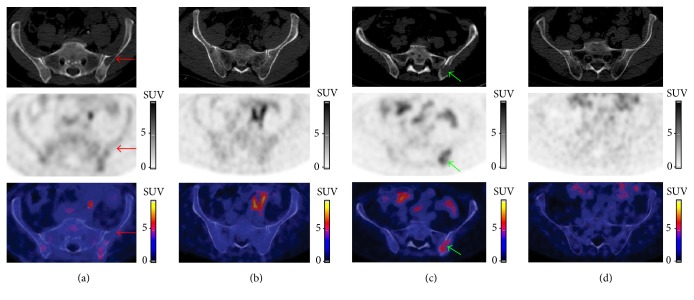
[^18^F]FPRGD_2_ PET/CT images of all patients. A diffuse bone marrow involvement was described based on CT images in patients #1 (a) and #2 (b); the [^18^F]FPRGD_2_ PET images also showed diffuse and heterogeneous bone marrow [^18^F]FPRGD_2_ uptake in patients #1 (a) and a mild diffuse bone marrow uptake in patient #2 (b). In contrast, no diffuse bone marrow [^18^F]FPRGD_2_ uptake was seen in patients #3 ((c) the green arrows point at a lytic FL with [^18^F]FPRGD_2_ uptake) and #4 ((d) no lesion shown). Note that, in patient #1 (a) with newly diagnosed MM, a large osteolytic FL in CT images did not show [^18^F]FPRGD_2_ uptake above the bone marrow background ((a) red arrows).

Our purpose was to explore the detection capabilities of [^18^F]FPRGD_2_ PET/CT and to assess its feasibility in MM disease. In the studied patients, the detection rate of [^18^F]FPRGD_2_ PET was lower than the detection rate of low dose CT alone (Figure 1). Every FL showing [^18^F]FPRGD_2_ uptake corresponded to an osteolytic lesion or a fracture on low dose CT images. Although the integrin *α*_*v*_*β*_3_ is expressed by multiple cells in tumor microenvironment such as MM tumor cells, osteoclasts, and activated endothelial cells during angiogenesis, our clinical observation suggests that [^18^F]FPRGD_2_ PET/CT does not allow a higher detection rate of MM bone lesions than low dose CT alone. The detection rate of [^18^F]FPRGD_2_ PET was overall lower than [^18^F]NaF/[^18^F]FDG PET (patient #3; Figures 1 and 3) but in one patient, more lesions were visible on the [^18^F]FPRGD_2_ scan (patient #1; Figures 1 and 2). The prognostic value of [^18^F]FPRGD_2_ positive lesions and the value of [^18^F]FPRGD_2_ PET/CT in patients with asymptomatic disease (and thus without bone lesions) were not studied and could be of interest. On the other hand, the high bone marrow background activity related to [^18^F]NaF uptake may explain why some of the FLs detected with [^18^F]FPRGD_2_ PET/CT were not seen with [^18^F]NaF/[^18^F]FDG. Diffuse bone marrow infiltration was not reliably estimated with [^18^F]NaF/[^18^F]FDG PET due to high [^18^F]NaF bone uptake while it was suspected with [^18^F]FPRGD_2_ PET/CT in 2 of the 4 patients (Figure 4).

Our report included 2 patients with relapsed MM and thus with possible long-lasting healed lesions. In one of these patients (patient #3; Figure 3), some of the osteolytic lesions did not show uptake of [^18^F]FPRGD_2_ while [^18^F]NaF/[^18^F]FDG PET showed tracer's uptake in all these lesions, indicating residual activity. However, whether the uptake was related to [^18^F]FDG in the presence of residual metabolically active tumor and/or whether it was related to [^18^F]NaF due to bone turnover in the long-lasting healing process of bone lesions after treatment is unknown [21]. Moreover, we excluded patients with a short treatment-free interval before inclusion to avoid PET-negativity induced by a recent chemotherapy.

As mentioned in the introduction, both imaging techniques highlight different biological aspects. [^18^F]FPRGD_2_ allows the estimation of integrin *α*_*v*_*β*_3_ expression by endothelial cells (and thus neovascularization), tumor cells, and activated osteoclasts, while [^18^F]NaF/[^18^F]FDG uptake reflects tumor cell metabolism and/or bone formation. The heterogeneous uptake of [^18^F]FPRGD_2_ can be explained by biological phenomena and previously received treatments. The myeloma-induced angiogenesis appears after an “angiogenic switch” due to the release of angiogenic factors by subsets of myeloma cells or can be directly in proportion to the tumor infiltration inside the bone marrow [22]. This angiogenesis is counteracted by targeted treatments such as thalidomide and bortezomib which could explain reduced uptake in relapsing patients. Decreased uptake of [^18^F]FDG was recently found to be associated with reduced expression of* hexokinase-2, *responsible for the first step of glycolysis [23].

Even though this case report suggests that [^18^F]FPRGD_2_ PET/CT might not be appropriate for detection of MM lesions, it may be of use in the assessment of integrin *α*_*v*_*β*_3_ expression in MM lesions, especially in clinical trials evaluating inhibitors targeting *α*_*v*_*β*_3_ integrins, as recently investigated by Tucci et al. [24]. In addition, our study focused on patients with symptomatic myeloma disease, while [^18^F]FPRGD_2_ PET/CT might be useful to detect bone marrow infiltration in precursor states of the disease (smoldering multiple myeloma or monoclonal gammopathy of undetermined significance).

## 5. Conclusions

In this case report, [^18^F]FPRGD_2_ PET/CT detected only 50% of the FLs detected by CT suggesting that the clinical utility of [^18^F]FPRGD_2_ PET/CT is rather limited for the detection of overt MM lesions. However, the clinical and possibly prognostic relevance of [^18^F]FPRGD_2_ positive MM lesions needs further investigation.

## Figures and Tables

**Table 1:** Patients' characteristics (*n* = 4).

Feature	*n*
Median age (range)	
65 (51–79) years	4
Sex	
Female	1
Male	3
Mean ± SD BMPC infiltration (%)	
48 ± 29%	4
Ig isotype	
IgG	4
ISS stage at diagnosis	
I	1
III	3
Relapsed MM	
*Time from diagnosis*	2
40 & 58 months	
*Time from last treatment*	
35 & 52 months	
Prior treatment	
Thalidomide-dexamethasone/ASCT	1
Melphalan-prednisone-thalidomide	1
No prior bisphosphonates therapy	4

Ig = immunoglobulin; BMPC = bone marrow plasma cell; ASCT = autologous stem cell transplantation.

**Table 2:** Focal lesions detected with CT and PET and lesions' characteristics.

	Whole-body	[^18^F]FPRGD_2_	[^18^F]NaF/[^18^F]FDG
	CT	PET	PET
Number of osteolytic lesions (*n* = 72)	*n* = 72 (89%)	*n* = 36 (44%)	*n* = 47 (64%)^††^
*Mean ± SD SUV*_*max*_		*2.5 ± 0.8*	*8.5 ± 4.3*
Number of fractures (*n* = 8)	*n* = 8 (10%)	*n* = 8 (10%)	*n* = 8 (10%)
*Mean ± SD SUV*_*max*_		*3.3 ± 1.2*	*9.4 ± 2.3*
Number of FLs without any abnormality on CT images	*n* = 0	*n* = 0	*n* = 1 (1%)

Total number of FLs (*n* = 81)^†^	*n* = 80 (99%)	*n* = 44 (54%)	*n* = 56 (69%)

^†^Number of FLs regardless of the presence of bone abnormality on low dose CT images, or hypoactive FLs with underlying bone destruction on CT images were considered PET FLs. ^††^Two out of 47 were hypoactive FLs; they were not considered in the measurement of SUV.

**Table 3:** Focal lesions detected per patient.

Patient	Newly diagnosed MM		Relapsed-MM		Total
	# 1	# 2	# 3	# 4	
Concordant results^†^	2	1	28	0	*31*
[^18^F]FPRGD_2_+ and [^18^F]NaF/FDG−	5	0	0	0	*5*
[^18^F]FPRGD_2_− and [^18^F]NaF/FDG+	3	0	12	1	*16*
[^18^F]FPRGD_2_ & CT− and [^18^F]NaF/FDG+	0	0	1	0	*1*
CT− and [^18^F]FPRGD_2_+	0	0	0	0	*0*
CT+ and both PET−	8	2	10	0	*20*
*Total malignant lesions*	*18*	*3*	*51*	*1*	*73*
Fractures	3	3	1	1	8

^†^Osteolytic FLs showing both [^18^F]FPRGD_2_ and [^18^F]NaF/FDG  uptake.

## Abbreviations

PET: Positron emission tomography
CT: Computed tomography
[^18^F]FDG: [^18^F]Fluorodeoxyglucose
FL: Focal lesion
MM: Multiple myeloma
WB: Whole-body
IMWG: International Myeloma Working Group
SUV: Standardized uptake value
SD: Standard deviation.
